# Ruminal ciliates as modulators of the rumen microbiome

**DOI:** 10.5713/ab.23.0309

**Published:** 2023-12-29

**Authors:** Tansol Park

**Affiliations:** 1 Department of Animal Science and Technology, Chung-Ang University, Anseong 17546, Korea

**Keywords:** Feed Efficiency, Genomics, Methane Mitigation, Nitrogen Utilization Efficiency, Ruminal Ciliates, Transcriptomics

## Abstract

Ruminal ciliates are a fundamental constituent within the rumen microbiome of ruminant animals. The complex interactions between ruminal ciliates and other microbial guilds within the rumen ecosystems are of paramount importance for facilitating the digestion and fermentation processes of ingested feed components. This review underscores the significance of ruminal ciliates by exploring their impact on key factors, such as methane production, nitrogen utilization efficiency, feed efficiency, and other animal performance measurements. Various methods are employed in the study of ruminal ciliates including culture techniques and molecular approaches. This review highlights the pressing need for further investigations to discern the distinct roles of various ciliate species, particularly relating to methane mitigation and the enhancement of nitrogen utilization efficiency. The promotion of establishing robust reference databases tailored specifically to ruminal ciliates is encouraged, alongside the utilization of genomics and transcriptomics that can highlight their functional contributions to the rumen microbiome. Collectively, the progressive advancement in knowledge concerning ruminal ciliates and their inherent biological significance will be helpful in the pursuit of optimizing rumen functionality and refining animal production outcomes.

## INTRODUCTION

The rumen is a complex anaerobic digestive ecosystem, which is present in ruminant animals, and plays a critical role in the digestion and fermentation of ingested diets, including starch, fiber contents, etc. [1]. This anaerobic digestion depends entirely on an array of microbial communities, including ruminal bacteria, archaea, fungi, and ciliated protozoa [2]. Among these members, ruminal ciliates comprise up to 50% of the microbial biomass within the rumen [3,4]. Their distinct biological roles, which encompass the predation on fellow ruminal microbes, the removal of oxygen within the rumen, and symbiotic relationships with prokaryotes—particularly hydrogenotrophic methanogens—have profound impacts on both ruminal functions and the overall performance of the host animals [4,5]. Notably, ruminal ciliates play a pivotal role in feed digestion, consistently demonstrating significantly higher digestibility across diverse measurements [6]. However, within the spectrum of the digestion processes, especially those potentially linked to global warming and arise from ruminant production, ruminal ciliates are recognized contributors and account for 9% to 37% of the ruminal methane production through direct or indirect associations with ruminal methanogens [7–9]. Additionally, ruminal ciliates contribute to excessive nitrogen recycling within the rumen, paralleling the limited nitrogen utilization efficiency (NUE) observed in host ruminants [10,11].

The composition of ruminal ciliates varies based on factors such as geographic distribution, diet, and among various animal species; thus, has led to a range of between 2 and 45 identified species [12]. Baraka, 2012 observed up to 12 distinct genera per ruminant breed and provided detailed visual descriptions and figures of 54 identified ciliate species [13]. Interestingly, substrate preferences, host specificity, and antagonistic interactions between ciliate species create four fauna types: type A (characterized by the presence of *Polyplastron* and *Ophryoscolex*), type B (featuring *Epidinium* and *Eudiplodinium*), type K (involving *Elytroplastron bubali*), and type O (consisting solely of *Entodinium* and isotrichids). These categories were proposed by Dehority [12] and Imai et al [14,15], although mixed populations have also been previously documented [16,17].

A prior study delving into the core microbiota through partial 18S rRNA gene sequencing demonstrated that 12 genera of ruminal ciliates account for nearly 100% relative abundance globally with substantial compositional variations, even among co-located animals [18]. Moreover, the functionally diverse nature of ruminal ciliate species complicates interpretation, particularly within the context of ongoing microbiome investigations, which often depend on morphology or marker genes. Moreover, the absence of a reliable reference database underscores the significance of unraveling the biological functions and metabolic contributions of individual ciliate species. Broadly, the population of ruminal ciliates can be delineated into starch-preferring species (e.g., *Entodinium*, [4]), lovers of simple sugars (e.g., isotrichids, [19]), cellulolytics (e.g., *Epidinium*, *Ostracodinium*, *Eudiplodinium*, and *Ophryoscolex*, [20]), and voracious bacterivores (e.g., *Entodinium* spp. and Diplodiniinae, [21,22]). However, these classifications necessitate validation through genomic or transcriptomic exploration, given that much of the knowledge surrounding ruminal ciliates stems from indirect evidence, such as defaunation and *in vitro* monocultures. Despite numerous endeavors aimed at comprehending the roles, biology, and contributions of ruminal ciliates on ruminal microbiome functions, notable gaps remain in both knowledge and technology.

### Culture-based methods for investigating ruminal ciliates

Significant challenges remain in the study of ruminal ciliates, such as achieving axenic cultures of these microorganisms in laboratory settings, which so far has proven elusive [23–25]. A compelling consensus has emerged that the growth of ruminal ciliates, even if not entirely axenic, depends on the presence of live microbes, particularly bacteria [26]. Consequently, instead of pursuing axenic cultures, researchers have extensively employed monoxenic cultures, which are cultures where a specific type of ruminal ciliate coexists with associated or free-living microbes, using *in vitro* conditions to facilitate the study of ruminal ciliates.

The feasibility of studying individual ruminal ciliate species largely relies on their amenability to cultivation. The successful maintenance of various entodiniomorphids in laboratory cultures has been reported, including for species such as *Diploplastron affine* [27], *Entodinium* spp. [27–31], *Epidinium* sp. [30,31], *Enoploplastron triloricatum* [30], *Eudiplodinium* spp. [27,32], and *Ophryoscolex purkynjei* [32]. A study by Dehority proposed the use of two basal medium compositions for culturing ruminal ciliates, involving *Entodinium* and *Epidinium* species, which were combined with 10% (v/v) clarified rumen fluid [33]. Additionally, chemically defined culture media have been formulated for ruminal ciliates [31]. However, maintaining long-term isotrichids cultures, such as the two major genera, *Isotricha* and *Dasytricha*, has proven challenging when using *in vitro* conditions [19]. Consequently, most research involving these genera employs monofaunated animals [11,34,35]. The intricate chemotactic behaviors of these sugar-loving ciliates, coupled with their inability to self-regulate their appetite, make closed batch culture systems less ideal [19,36]. Since the practical maintenance of monofaunated ruminants poses difficulties, ongoing efforts to enhance the culturability of diverse ruminal ciliates remain valuable for expanding knowledge and investigating their responses to dietary interventions and feed additives *in vitro*.

Initially, single-cell isolation was employed to establi sh monocultures of ruminal ciliate species [33] and to analyze ciliate-associated prokaryotes [16,37]. Serial dilution (or transfer) in a sterile medium during single-cell isolation serves to exclude as many free-living microbes as possible, thereby facilitating detailed analysis of ciliate-associated microbes. For comprehensive microbiome analysis, the utilization of high-throughput cell sorting methods can enhance the number of isolated cells, yielding more informative specimens for microbial association studies.

Ruminal ciliates exhibit a wide range of cell sizes, typically spanning 10 to 200 μm, based on cell length [4]. Thus, this size diversity has prompted ruminal microbiologists to explore ecological distinctions based on size-based ciliate fractions [38]. Williams and Coleman, 1992 furnished fundamental guidelines for size-based fractionation of individual or grouped ruminal ciliates, thereby offering an initial approach for isolating ciliate species of interest [4]. Recently, marker gene-based sequencing approaches have confirmed the presence of differentially abundant bacterial taxa (primarily within Proteobacteria) in ciliate-associated fractions facilitated by both single-cell isolation [16] and size-based fractionations [39].

In addition to the use of appropriate washing procedures, electron microscopy (including scanning and transmission electron microscopy) supplemented by fluorescence staining can visually corroborate the physical associations between ruminal ciliates and their potential symbionts [40,41]. Thus, if necessary, the use of antioxidant mixtures [42] and stepwise freezing procedures [34] can enhance the storage of ruminal ciliate cultures during long-term freezing.

### Molecular-based approaches for classifying and enumerating ruminal ciliates (metataxonomics)

While absolute cell counts of ruminal ciliates have traditionally been used to assess their contributions within experimental trials, the compositional analysis of these populations offers valuable insights into their functional diversity. Microscopic identification of ruminal ciliates, a technique employed for decades, requires expertise and references to established works [12,13,43,44]; thus, specific morphological differentiation of individual species remains challenging.

In parallel with the gradual replacement of quantitative real-time polymerase chain reaction by amplicon sequencing for bacterial and archaeal population analyses, 18S rRNA gene sequencing has been considered an alternative for quantifying ruminal ciliates. However, due to the 18S rRNA gene being highly conserved among ciliate genera or species, coupled with the limited availability of full-length reference sequences in public databases [45], the use of molecular-based enumeration for ruminal ciliates still encounters limitations.

The current classification of 18S rRNA gene sequences, via BLASTn against reference databases, is only accessible in a standard nucleotide database for ruminal ciliates classification (accessed on 2023.08.13). BLAST matches can provide taxonomy information down to the species level. Nonetheless, short reads (e.g., 200 to 300 bp) typically yield reliable family- or genus-level classifications, owing to duplicated matches and associations with numerous unclassified ciliate sequences (data not presented). Silva offers high-quality ribosomal RNA databases, including over 2,000 reference 18S rRNA gene sequences from at least 15 genera of ruminal ciliates ([46], accessed on 2023.08.12; Table 1). However, when applying Silva reference databases (latest version, v138) within the QIIME2 environment for ciliate sequence classification, many short amplicon sequence variants cannot be assigned any genus name with default confidence scores (data not presented). Consequently, especially when dealing with short reads, careful interpretation of taxonomy classification is warranted. Furthermore, employing long-read sequencing with contemporary platforms that target robust phylogenetic markers, such as the internal transcribed spacer DNA or 28S rRNA gene sequences, is recommended for more reliable metataxonomic analysis of ruminal ciliates.

### Methane mitigation strategies in the context of ruminal ciliate populations

Ruminal ciliates are known to produce hydrogen through carbohydrate fermentation [8]. Notably, interspecies hydrogen transfer has emerged as a pivotal reason for the elevated methane production associated with ruminal ciliates [47,48]. Moreover, this insight led Guyader et al [48] to unveil a robust positive correlation between ruminal ciliate cell counts and methane production, which was backed by their meta-analysis of 28 experiments. Direct evidence of this interaction was provided from co-cultivation experiments, involving *Polyplastron multivesiculatum* and *Methanosarcina barkeri*, which revealed significantly higher methane production accompanied by hydrogen uptake within the co-culture [49]. Remarkably, this specific association accounted for up to a third of the total methane production [7–9], whereas complete removal of ruminal ciliates led to a reduction in methane output [6]. Furthermore, it is worth noting that partial inhibition of ruminal ciliates also had a mitigation effect on methane production [50], thereby underscoring the significance of curtailing ciliate-associated methanogenic populations for effective methane mitigation.

Fractionation-based investigations have highlighted dif ferences in the methanogen population between free-living and ciliate-associated fractions [51]. For instance, *Methanobrevibacter* spp., are proportionally more associated with the ciliate-associated fraction, which is a phenomenon attributed to specific adhesins produced by the genus [52]. Moreover, within the *Methanobrevibacter* species, the prevalence of the SGMT clade in cattle has been linked to higher methane production [53–55]. Although research concerning the relationship between this clade and ruminal ciliates is limited, strategies targeting this specific methanogen–ruminal ciliate association could be promising for methane reduction. However, further research is needed to ascertain the specificity of this association pattern.

Methane-mitigating feed additives can have direct or in direct impacts on ruminal ciliate populations and are a critical factor influencing the success or failure of these applications in animal trials. The practical application of 3-NOP directly inhibited methanogenesis and resulted in an increase in hydrogen concentration; however, it failed to demonstrate the suppression of hydrogen-producing microorganisms, including ruminal ciliates, in sheep [56], dairy [57,58], and beef cattle [59] trials. This could be attributed to the intricate nature of the anaerobic microbiome and hydrogen balance in the rumen [60]. However, higher doses did achieve sufficient hydrogen accumulation required to inhibit certain ruminal ciliate species, which resulted in a reduction in fibrolytic activity [60,61]. Nevertheless, the results are mixed, and while ruminal ciliate contributions to nitrate reduction have been noted during methane mitigation, an understanding of the mechanisms involved remains incomplete. Further, an enhanced metabolic reduction in nitrate and nitrite has been observed in faunated conditions in both *in vitro* and *in vivo* studies [62], with ciliates and associated bacteria potentially contributing to nitrite reduction [63]. This interaction highlights the role of ruminal ciliates in ensuring the safe application of nitrate for methane mitigation. Natural-product-based methane inhibitors, such as essential oil blends, endeavor to amplify efficacy through the combination of diverse plant secondary metabolites. Commercial products, such as Agolin and Mootral have demonstrated significant methane reduction in sheep [64] and dairy cattle [65], albeit with limited effectiveness in beef cattle [66]. Highly permeable plant secondary metabolites could potentially influence ruminal ciliate and methanogen populations [67], although a meta-analysis suggested inconsistent effects on ruminal ciliates reduction through essential oil blends [68]. Saponins and tannins, naturally occurring secondary metabolites in plants, have long been recognized as methane inhibitors that potentially target both hydrogen-producing microorganisms and methanogens [69–71]. A meta-analysis of *in vitro* experiments indicated that saponin supplementation significantly reduced total ciliate concentrations, coupled with moderate methane mitigation effects [72]. Additionally, the analysis of 49 sheep studies suggested that methane was significantly reduced through dietary saponins, based on methane yield per dry matter intake [73]. Among the *in vivo* studies reporting on ruminal ciliates in saponin applications, two-thirds observed a decrease in the total ciliate counts [74]. A recent meta-analysis involving beef cattle and tannin supplementation supported the methane mitigation potential, with a trend toward reduced ciliate counts [75]. The anti-protozoal activity of these representative plant secondary metabolites has been acknowledged, yet the extraction methods, dosages, plant source origins, and target animals should be considered to definitively ascertain their anti-protozoal activities while pursuing methane mitigation in ruminants.

In total, several methane inhibitors incidentally inhibited protozoal activity, at least partially. However, total ciliate counts have primarily been employed as representative measurements to analyze their contribution to methane production. Notably, different groups of ruminal ciliates, namely entodiniomorphids and isotrichids, exhibit varying contributions to methane production in the rumen due to disparities in hydrogen production abilities [76]. Their different methanogen associations have also been observed [35], as demonstrated in a study on transfaunation involving ciliate-free sheep inoculated with isotrichids [77]. Given their mutualistic roles with methanogens, these interactions could differ based on the ciliate species involved. Therefore, for synergistic or consistent methane reduction in the intricate rumen environment, the mechanisms of methane inhibitors on overall ruminal ciliate populations warrant further detailed investigations.

### The influence of ruminal ciliate populations on the nitrogen utilization efficiency

Previous studies have shown that ruminal ciliates harbor an array of protease-coding genes [78,79], and have also confirmed their proteolytic activity [80,81]. Although the presence of deaminase activity is yet to be confirmed through genome- and activity-based investigations, ruminal ciliates have been associated with a negative impact on NUE [82].

Despite the fact that ruminal ciliate proteins possess an amino acid composition, which is preferable for the host animal than bacterial proteins [83], their actual contribution as microbial proteins to the host is likely less than their numerical presence in the rumen; thus, can ultimately be attributed to their behavioral traits including sequestration in the rumen [84,85] and overestimated ciliate proteins by bacterial contamination [86].

Moreover, efforts to enhance NUE in the rumen have tar geted the ruminal ciliate population through complete (i.e., defaunation) or partial inhibition approaches. A 2015 meta-analysis of 23 *in vivo* studies employing defaunating agents or methods in sheep and cattle underscored the significant negative impact of ruminal ciliates on NUE-related measurements. This effect extended to both ruminal measurements, such as ammonia concentration and efficiency of microbial protein synthesis, and to measurements reflecting nitrogen flow in the lower gut, including duodenal nitrogen flow and urinary nitrogen excretion [6]. These findings align with earlier research by Eugène et al [87]. In 2019, Dai and Faciola [50] expanded this investigation to include 50 partial inhibition studies utilizing phytochemicals and lipids and concluded that reducing the ruminal ciliate population, notably, improved the ruminal ammonia nitrogen concentration.

Bacterivory activity varies among ruminal ciliate families or genera [21]. Therefore, considering both bacterivory activity and ciliate cell counts, *Entodinium* and *Diplodinium* spp. emerged as the potential factors limiting NUE [21]. However, the specific effects of these ciliate groups may vary with factors such as age, diet, host breeds, and genetics. A study involving monofaunated sheep demonstrated that the presence of ruminal ciliates resulted in a reduced transfer of microbial protein into the duodenum, with this effect being more pronounced in *Entodinium*-monofaunated sheep [11,88].

The diverse impacts of nitrogen recycling associated with distinct ciliate populations have encouraged researchers to explore specific ciliate inhibition strategies. Given that ruminal ciliates enhance the overall ruminal digestibility through their activities and the indirect neutralization of the ruminal environment [6], achieving an improved NUE by adjusting the ruminal ciliate population, particularly through partial inhibition of bacterivory ciliate species, is an appealing approach. However, further investigation is required. *In vitro* studies utilizing both ciliate monocultures and rumen fluid inocula have shown that restraining protease activities, while not limited to ruminal ciliates, could enhance NUE without adverse effects on ruminal fermentation and digestion [89,90]. Approaches such as selecting target enzymes specific to ruminal ciliates, screening feed additives with anti-protozoal properties, and adjusting rumen passage rates are also worthy of consideration.

### The potential influence of ruminal ciliates on feed efficiency

Numerous research efforts have been directed towards elucidating the factors underpinning the variations in the host feed efficiency and are often quantified using residual feed intake (RFI), within the context of the rumen microbiome. Thus, the lower microbial and functional diversities observed in the rumen microbiome potentially enable more efficient dietary energy utilization [91]. Notably, some potential microbial biomarkers have been consistently identified in both low- and high-RFI ruminant groups. However, limited attention has been paid to the role of ruminal ciliates in this context. The meta-analysis mentioned earlier, focused on the impact of defaunation and established a connection between ruminal ciliates and higher dry matter intake, which resulted in lower average daily gains, and consequently, reduced feed conversion efficiency; a result that was particularly evident in low-quality diets [6,87]. Despite the specific effects of individual ciliate species on each aspect of animal performance not yet having been defined, these limitations are likely due to the constrained NUE and increased metabolic heat production, which can potentially counteract the positive effect of ruminal ciliates on overall digestibility [6].

In the case of Angus steers, the overall community of ru minal ciliates differed according to RFI [92]. Although no differences were observed among the classified ciliate genera in relation to RFI, efficient Angus steers exhibited higher operational taxonomic unit richness and phylogenetic diversity in their ciliate communities. These characteristics in low-RFI animals might facilitate an increase in feed digestibility and energy extraction from substrates. The intriguing phenomenon of preferential bacterivory on specific bacterial groups, especially major cellulolytic consortia, could potentially arise due to unbalanced dietary preferences among individual ciliate species. However, this hypothesis remains inconclusive due to the limited number of prey tested to date [93,94].

Moreover, attempts to enhance the overall animal perfor mance through ruminal ciliates-enriched inocula, particularly for entodiniomorphids, have currently failed to yield significant improvements, both in pre-weaning dairy calves [95,96] and in post-weaning periods [97]. Although the positive impact on diarrhea reduction was not linked to shifts in the rumen microbiome or ciliate populations, more comprehensive studies are warranted to assess the advantages and disadvantages of ruminal ciliates, particularly for young ruminants.

Furthermore, the composition of ruminal ciliates could potentially influence the enrichment of bacterial groups associated with feed efficiency. Differential abundance by Proteobacteria in ciliate-associated fractions has been observed in various rumen studies [16,37–39], as well as in marine ciliates [98]; a correlation that might be connected to the prevalence of Succinivibrionaceae in feed-efficient animals [99,100]. Given that these bacteria are known hydrogen utilizers, their affiliation with ruminal ciliates seems metabolically sound, enabling them to outcompete other bacterial rivals within the faunated environment. Consequently, the reported association between ruminal ciliates and rumen microbiota markers within the feed efficiency context should be further explored to facilitate their enrichment within the rumen.

### Future prospects in the study of ruminal ciliates: genomics and transcriptomics

As highlighted earlier in this review, the inability to culture axenic populations of ruminal ciliates presents a significant challenge, limiting the application of conventional microbiological methods to characterize these unculturable unicellular eukaryotes. Therefore, much of our understanding of ruminal ciliates stems from indirect evidence, including defaunation studies or *in vitro* cultures encompassing other domains of rumen microbes [6,23,24,29,101]. To transcend the constraints of culture-based approaches, sequencing the metabolically active macronuclear genome offers authentic insights into ruminal ciliates, which has been aided by advancements in sequencing technologies. The initial macronuclear genome sequencing of *Entodinium caudatum* has paved the way for future research into fundamental questions on ruminal ciliates and the exploration of practical strategies for manipulating these ruminal ciliates within the rumen [78]. This genome, complemented by previous transcriptomic data [102], has unveiled evidence of horizontal gene transfers of numerous carbohydrate-active enzymes (CAZymes) and an abundance of protease-coding genes. These findings underscore the adaptive strategy of the ruminal ciliates for surviving in a carbohydrate-rich and complex anaerobic microbiota in the rumen environment. Building on these insights, Li et al [79] extended the exploration by adding 69 single-cell amplified genomes from ruminal ciliates to the repertoire. Their assembly of telomereless contigs from macronuclear sequences has facilitated an approximate 12% average mapping rate for metagenomic reads from publicly available rumen metagenomes to ciliate genomes. Hence, single-cell sequencing has emerged as a compelling avenue for studying ruminal ciliates, which are often contaminated with other microbes and are resistant to culturing. However, owing to technical constraints in multiple displacement amplifications, which is employed for isolated single cells, overall genome completeness ranged from 8% to 91%. This wide range underscores the need for improved sequencing platforms and a greater repository of ciliate culture collections to refine these tentative ciliate genomes. As these future genome databases mature, the development of genetic tools tailored to ruminal ciliates is essential for conducting functional genomics that validates the roles of genes annotated within the ciliate genome. Single-cell transcriptomics, complemented by precise cell sorting, is a powerful tool for analyzing differential gene expression profiles of individual ciliate cells under diverse feeding and environmental conditions, uncontaminated by prokaryotic data.

The application of biochemical characterizations following macronuclear genome sequencing holds promise in verifying the activity of protein-coding genes, particularly CAZymes of ruminal ciliates. Findley et al [103] and Williams et al [104] utilized an activity-based screening from cDNA libraries of ruminal ciliates-enriched samples to successfully characterize carbohydrate-degrading enzymes, thereby demonstrating the degradation of plant cell wall constituents. Additionally, Williams et al [104] also used a metatranscriptomic analysis to provide evidence of fungal predation by ruminal ciliates, thereby revealing elevated chitinase expression. To effectively implement these approaches, as emphasized by these authors, it is essential to enrich for polyadenylated mRNA in order to eliminate microbiota contamination before cDNA synthesis followed by the concentration of targeted ruminal ciliates.

## CONCLUSION

Collectively, the research presented in this review has attempted to advance our understanding of ruminal ciliates, particularly in elucidating their complex associations with prokaryotic partners, their contributions to ruminal metabolism, and the modulation of the ruminal microbiome. Recent meta-analyses have highlighted the relationship between ruminal ciliates and both ruminal methanogenesis and NUE. Within the dynamic framework of the ruminal microbiome, there exists a compelling need for systematic investigations, which are focused on delineating the precise interactions between ruminal methanogens and deaminating bacteria, a crucial avenue that can be used to steer ruminal fermentation to environmentally sustainable outcomes. To achieve this crucial goal, it becomes imperative to develop streamlined and reproducible cultivation methods and to elevate molecular approaches capable of discerning prokaryotic contaminants within the complex consortium of ruminal ciliates. Furthermore, the momentum placed on refining these methodologies is important because it will allow for more rigorous investigations. In this way, the integration of more comprehensive genomic insights for individual ruminal ciliate species is a most welcome development. Indeed, these robust genome resources will furnish bioinformatic reference databases, serving as both markers for taxonomic classification and as critical tools for precise functional annotation in the broader analysis of the entire rumen microbiome. Guided by these multifaceted approaches, the field of ruminal ciliate research is poised to increase our comprehension of this interesting microbial cohort. Moreover, as we continue to investigate their basic biology and interplay with other ruminal microorganisms, the way of ruminal ciliate investigations is primed to guide in a new era of understanding.

## Figures and Tables

**Table 1:** Small subunit rRNA gene sequences from ruminal ciliates, deposited on Silva databases (v138)^1),2)^

Genus	No. of deposited sequences	Sequence length statistics (bp)			Avg. sequence quality (%)	Species

		Avg.	Min	Max		
*Charonina*	16	1,316	806	1,537	94.25	*Charonina ventriculi*
*Dasytricha*	592	1,160	333	1,638	92.44	*Dasytricha ruminantium*
*Diplodinium*	141	1,299	464	1,780	94.45	*Diplodinium anisacanthum, D. dentatum*, and *D. denticulatum*
*Diploplastron*	29	1,011	465	1,637	94.62	*Diploplastron affine*
*Enoploplastron*	2	1,638	1,637	1,639	92.86	*Enoploplastron triloricatum*
*Entodinium*	688	1,170	317	1,647	94.54	*Entodinium bursa, E. caudatum, E. dubardi, E. furca, E. longinucleatum, E. nanellum*, and *E. simplex*
*Eodinium*	4	1,444	1,436	1,457	91.63	*Eodinium posterovesiculatum*
*Epidinium*	13	1,290	693	1,638	94.04	*Epidinium caudatum*, and *E. ecaudatum caudatum*
*Eremoplastron*	21	1,358	465	1,636	94.43	*Eremoplastron dilobum*, and *E. rostratum*
*Eudiplodinium*	108	1,403	346	1,637	93.68	*Eudiplodinium maggii*
*Isotricha*	132	904	400	1,641	90.80	*Isotricha intestinalis*, and *I. prostoma*
*Metadinium*	29	899	465	1,637	92.52	*Metadinium medium*, and *M. minorum*
*Ophryoscolex*	272	1,111	464	1,636	92.92	*Ophryoscolex caudatus*, and *O. purkynjei*
*Ostracodinium*	16	1,489	1,362	1,639	93.37	*Ostracodinium clipeolum, O. dentatum, O. gracile, O. rugoloricatum*, and *O. trivesiculatum*
*Polyplastron*	281	1,256	330	1,639	93.52	*Polyplastron multivesiculatum*
Uncultured	18	571	464	1,364	96.24	-
Total	2,362	1,171	317	1,780	93.42	-

1) Accessed on 2023.8.12.

2) Sequences from non-ruminal ciliates were not included.

## References

[1] Huws SAH, Creevey C, Oyama LB (2018). Addressing global ruminant agricultural challenges through understanding the rumen microbiome: past, present and future. Front Microbiol.

[2] Mizrahi I (2013). Rumen symbioses. The Prokaryotes.

[3] Leng RA, Nolan JV (1984). Nitrogen metabolism in the rumen. J Dairy Sci.

[4] Williams AG, Coleman GS (1992). The rumen protozoa. Springer Series in Contemporary Bioscience.

[5] Bonhomme A (1990). Rumen ciliates: their metabolism and relationships with bacteria and their hosts. Anim Feed Sci Technol.

[6] Newbold CJ, de la Fuente G, Belanche A, Ramos-Morales E, McEwan NR (2015). The role of ciliate protozoa in the rumen. Front Microbiol.

[7] Finlay BJ, Esteban G, Clarke KJ, Williams AG, Embley TM, Hirt RP (1994). Some rumen ciliates have endosymbiotic methanogens. FEMS Microbiol Lett.

[8] Newbold CJ, Lassalas B, Jouany JP (1995). The importance of methanogens associated with ciliate protozoa in ruminal methane production in vitro. Lett Appl Microbiol.

[9] Martin C, Morgavi DP, Doreau M (2010). Methane mitigation in ruminants: from microbe to the farm scale. Animal.

[10] Ivan M, Neill L, Forster R, Alimon R, Rode LM, Entz T (2000). Effects of Isotricha, Dasytricha, Entodinium, and total fauna on ruminal fermentation and duodenal flow in wethers fed different diets. J Dairy Sci.

[11] Ivan M (2009). Comparison of duodenal flow and digestibility in fauna-free sheep inoculated with holotrich protozoa, Entodinium monofauna or total mixed protozoa population. Br J Nutr.

[12] Dehority BA (2003). Rumen microbiology.

[13] Baraka T (2012). Comparative studies of rumen pH, total protozoa count, generic and species composition of ciliates in camel, buffalo, cattle, sheep and goat in Egypt. J Am Sci.

[14] Imai S (1978). Distribution of rumen ciliate protozoa in cattle, sheep and goat and experimental transfaunaiton of them. Nippon Chikusan Gakkaiho.

[15] Imai S, Katsuno M, Ogimoto K (1979). Type of the pattern of the rumen ciliate composition of the domestic ruminants and the predator-prey interaction of the ciliates. Nippon Chikusan Gakkaiho.

[16] Park T, Yu Z (2018). Do ruminal ciliates select their preys and prokaryotic symbionts?. Front Microbiol.

[17] Kišidayová S, Durkaj D, Mihaliková K (2021). Rumen ciliated protozoa of the free-living European bison (Bison bonasus, Linnaeus). Front Microbiol.

[18] Henderson G, Cox F, Ganesh S (2015). Rumen microbial community composition varies with diet and host, but a core microbiome is found across a wide geographical range. Sci Rep.

[19] Williams AG (1986). Rumen holotrich ciliate protozoa. Microbiol Rev.

[20] Coleman GS (1985). The cellulase content of 15 species of entodiniomorphid protozoa, mixed bacteria and plant debris isolated from the ovine rumen. J Agric Sci.

[21] Belanche A, de la Fuente G, Moorby JM, Newbold CJ (2012). Bacterial protein degradation by different rumen protozoal groups. J Anim Sci.

[22] Morgavi DP, Sakurada M, Tomita Y, Onodera R (1996). Electrophoretic forms of chitinolytic and lysozyme activities in ruminal protozoa. Curr Microbiol.

[23] Coleman GS (1962). The preparation and survival of almost bacteria-free suspensions of Entodinium caudatum. J Gen Microbiol.

[24] Hino T, Kametaka M (1977). Gnotobiotic and axenic cultures of a rumen protozoon, Entodinium caudatum. J Gen Appl Microbiol.

[25] Bonhomme A, Fonty G, Senaud J (1982). Attempt to obtain and maintain rumen entodiniomorph ciliates in axenic cultures. Ann Microbiol (Paris).

[26] Park T, Meulia T, Firkins JL, Yu Z (2017). Inhibition of the rumen ciliate Entodinium caudatum by antibiotics. Front Microbiol.

[27] Belzecki G, Miltko R, Kwiatkowska E, Michalowski T (2013). The ability of rumen ciliates, Eudiplodinium maggii, Diploplastron affine, and Entodinium caudatum, to use the murein saccharides. Folia Microbiol.

[28] Fondevila M, Dehority BA (2001). In vitro growth and starch digestion by Entodinium exiguum as influenced by the presence or absence of live bacteria. J Anim Sci.

[29] Fondevila M, Dehority BA (2001). Preliminary study on the requirements of Entodinium exiguum and E. caudatum for live or dead bacteria when cultured in vitro. Reprod Nutr Dev.

[30] Dehority BA (2008). Improved in vitro procedure for maintaining stock cultures of three genera of rumen protozoa. J Anim Sci.

[31] Kišidayová S, Váradyová Z, Zeleňák I, Siroka P (2000). Methanogenesis in rumen ciliate cultures of Entodinium caudatum and Epidinium ecaudatum after long-term cultivation in a chemically defined medium. Folia Microbiol.

[32] Dehority BA (2004). In vitro determination of generation times for Entodinium exiguum, Ophryoscolex purkynjei and Eudiplodinium maggii. J Eukaryot Microbiol.

[33] Dehority BA (1998). Generation times of Epidinium caudatum and Entodinium caudatum, determined in vitro by transferring at various time intervals. J Anim Sci.

[34] Nsabimana E, Kišidayová S, Macheboeuf D, Newbold CJ, Jouany JP (2003). Two-step freezing procedure for cryopreservation of rumen ciliates, an effective tool for creation of a frozen rumen protozoa bank. Appl Environ Microbiol.

[35] Belanche A, de la Fuente G, Newbold CJ (2014). Study of methanogen communities associated with different rumen protozoal populations. FEMS Microbiol Ecol.

[36] Williams AG (1979). The selectivity of carbohydrate assimilation by the anaerobic rumen ciliate Dasytricha ruminantium. J Appl Bacteriol.

[37] Irbis C, Ushida K (2004). Detection of methanogens and proteobacteria from a single cell of rumen ciliate protozoa. J Gen Appl Microbiol.

[38] Solomon R, Wein T, Levy B (2022). Protozoa populations are ecosystem engineers that shape prokaryotic community structure and function of the rumen microbial ecosystem. ISME J.

[39] Levy B, Jami E (2018). Exploring the prokaryotic community associated with the rumen ciliate protozoa population. Front Microbiol.

[40] Xia Y, Kong YH, Seviour R, Forster RJ, Kisidayova S, McAllister TA (2014). Fluorescence in situ hybridization probing of protozoal Entodinium spp. and their methanogenic colonizers in the rumen of cattle fed alfalfa hay or triticale straw. J Appl Microbiol.

[41] Valle ER, Henderson G, Janssen PH, Cox F, Alexander TW, McAllister TA (2015). Considerations in the use of fluorescence in situ hybridization (FISH) and confocal laser scanning microscopy to characterize rumen methanogens and define their spatial distributions. Can J Microbiol.

[42] Park T, Yu Z (2018). Aerobic cultivation of anaerobic rumen protozoa, Entodinium caudatum and Epidinium caudatum. J Microbiol Methods.

[43] Dehority BA (1993). Laboratory manual for classification and morphology of rumen ciliate protozoa.

[44] Imai S, Shinno T, Ike K, Morita T, Selim HM (2004). Fourteen morphotypes of Entodinium ovumrajae (Ophryoscolecidae, Entodiniomorphida) found in the Dromedary camel of Egypt. J Eukaryot Microbiol.

[45] Firkins JL, Yu Z, Park T, Plank JE (2020). Extending Burk Dehority’s perspectives on the role of ciliate protozoa in the rumen. Front Microbiol.

[46] Quast C, Pruesse E, Yilmaz P (2013). The SILVA ribosomal RNA gene database project: improved data processing and web-based tools. Nucleic Acids Res.

[47] Balch WE, Fox GE, Magrum LJ, Woese CR, Wolfe RS (1979). Methanogens: reevaluation of a unique biological group. Microbiol Rev.

[48] Guyader J, Eugène M, Nozière P, Morgavi DP, Doreau M, Martin C (2014). Influence of rumen protozoa on methane emission in ruminants: a meta-analysis approach. Animal.

[49] Ushida K, Newbold CJ, Jouany JP (1997). Interspecies hydrogen transfer between the rumen ciliate Polyplastron multivesiculatum and Methanosarcina barkeri. J Gen Appl Microbiol.

[50] Dai X, Faciola AP (2019). Evaluating strategies to reduce ruminal protozoa and their impacts on nutrient utilization and animal performance in ruminants-A meta-analysis. Front Microbiol.

[51] Tymensen LD, Beauchemin KA, McAllister TA (2012). Structures of free-living and protozoa-associated methanogen communities in the bovine rumen differ according to comparative analysis of 16S rRNA and mcrA genes. Microbiology.

[52] Ng F, Kittelmann S, Patchett ML (2016). An adhesin from hydrogen-utilizing rumen methanogen Methanobrevibacter ruminantium M1 binds a broad range of hydrogen-producing microorganisms. Environ Microbiol.

[53] Danielsson R, Schnürer A, Arthurson V, Bertilsson J (2012). Methanogenic population and CH4 production in Swedish dairy cows fed different levels of forage. Appl Environ Microbiol.

[54] Danielsson R, Dicksved J, Sun L (2017). Methane production in dairy cows correlates with rumen methanogenic and bacterial community structure. Front Microbiol.

[55] Smith PE, Kelly AK, Kenny DA, Waters SM (2022). Differences in the composition of the rumen microbiota of finishing beef cattle divergently ranked for residual methane emissions. Front Microbiol.

[56] Martínez-Fernández G, Abecia L, Arco A (2014). Effects of ethyl-3-nitrooxy propionate and 3-nitrooxypropanol on ruminal fermentation, microbial abundance, and methane emissions in sheep. J Dairy Sci.

[57] Haisan J, Sun Y, Guan LL (2014). The effects of feeding 3-nitrooxypropanol on methane emissions and productivity of Holstein cows in mid lactation. J Dairy Sci.

[58] Schilde M, von Soosten D, Hüther L, Meyer U, Zeyner A, Dänicke S (2021). Effects of 3-nitrooxypropanol and varying concentrate feed proportions in the ration on methane emission, rumen fermentation and performance of periparturient dairy cows. Arch Anim Nutr.

[59] Romero-Perez A, Okine EK, McGinn SM (2014). The potential of 3-nitrooxypropanol to lower enteric methane emissions from beef cattle. J Anim Sci.

[60] Morgavi DP, Forano E, Martin C, Newbold CJ (2010). Microbial ecosystem and methanogenesis in ruminants. Animal.

[61] Newbold CJ, López S, Nelson N, Ouda JO, Wallace RJ, Moss AR (2005). Propionate precursors and other metabolic intermediates as possible alternative electron acceptors to methanogenesis in ruminal fermentation in vitro. Br J Nutr.

[62] Villar ML, Hegarty RS, Clay JW, Smith KA, Godwin IR, Nolan JV (2020). Dietary nitrate and presence of protozoa increase nitrate and nitrite reduction in the rumen of sheep. J Anim Physiol Anim Nutr.

[63] Lin M, Schaefer DM, Guo WS, Ren LR, Meng QX (2011). Comparisons of in vitro nitrate reduction, methanogenesis, and fermentation acid profile among rumen bacterial, protozoal and fungal fractions. Asian-Australas J Anim Sci.

[64] Ahmed E, Batbekh B, Fukuma N (2021). A garlic and citrus extract: Impacts on behavior, feed intake, rumen fermentation, and digestibility in sheep. Anim Feed Sci Technol.

[65] Khurana R, Brand T, Tapio I, Bayat AR (2023). Effect of a garlic and citrus extract supplement on performance, rumen fermentation, methane production, and rumen microbiome of dairy cows. J Dairy Sci.

[66] Roque BM, Van Lingen HJ, Vrancken H, Kebreab E (2019). Effect of Mootral—a garlic-and citrus-extract-based feed additive— on enteric methane emissions in feedlot cattle. Transl Anim Sci.

[67] Ma T, Chen D, Tu Y (2016). Effect of supplementation of allicin on methanogenesis and ruminal microbial flora in Dorper crossbred ewes. J Anim Sci Biotechnol.

[68] Belanche A, Newbold CJ, Morgavi DP, Bach A, Zweifel B, Yáñez-Ruiz DR (2020). A meta-analysis describing the effects of the essential oils blend agolin ruminant on performance, rumen fermentation and methane emissions in dairy cows. Animals.

[69] Patra AK, Min BR, Saxena J, Patra A (2012). Dietary tannins on microbial ecology of the gastrointestinal tract in ruminants. Dietary phytochemicals and microbes.

[70] Cieslak A, Szumacher-Strabel M, Stochmal A, Oleszek W (2013). Plant components with specific activities against rumen methanogens. Animal.

[71] Patra A, Park T, Kim M, Yu Z (2017). Rumen methanogens and mitigation of methane emission by anti-methanogenic compounds and substances. J Anim Sci Biotechnol.

[72] Jayanegara A, Wina E, Takahashi J (2014). Meta-analysis on methane mitigating properties of saponin-rich sources in the rumen: influence of addition levels and plant sources. Asian-Australas J Anim Sci.

[73] Darabighane B, Mahdavi A, Aghjehgheshlagh FM, Navidshad B, Yousefi MH, Lee MRF (2021). The effects of dietary saponins on ruminal methane production and fermentation parameters in sheep: a meta analysis. Iranian J Appl Anim Sci.

[74] Patra AK, Saxena J (2009). Dietary phytochemicals as rumen modifiers: a review of the effects on microbial populations. Antonie Van Leeuwenhoek.

[75] Orzuna-Orzuna JF, Dorantes-Iturbide G, Lara-Bueno A, Mendoza-Martínez GD, Miranda-Romero LA, Hernández-García PA (2021). Effects of dietary tannins’ supplementation on growth performance, rumen fermentation, and enteric methane emissions in beef cattle: a meta-analysis. Sustainability.

[76] Paul RG, Williams AG, Butler RD (1990). Hydrogenosomes in the rumen entodiniomorphid ciliate Polyplastron multivesiculatum. Microbiology.

[77] Belanche A, de la Fuente G, Newbold CJ (2015). Effect of progressive inoculation of fauna-free sheep with holotrich protozoa and total-fauna on rumen fermentation, microbial diversity and methane emissions. FEMS Microbiol Ecol.

[78] Park T, Wijeratne S, Meulia T, Firkins JL, Yu Z (2021). The macronuclear genome of anaerobic ciliate Entodinium caudatum reveals its biological features adapted to the distinct rumen environment. Genomics.

[79] Li Z, Wang X, Zhang Y (2022). Genomic insights into the phylogeny and biomass-degrading enzymes of rumen ciliates. ISME J.

[80] Prins RA, Van Rheenen DL, van’t Klooster AT (1983). Characterization of microbial proteolytic enzymes in the rumen. Antonie van Leeuwenhoek.

[81] Forsberg CW, Lovelock LKA, Krumholz L, Buchanan-Smith JG (1984). Protease activities of rumen protozoa. Appl Environ Microbiol.

[82] Park T, Yu Z (2023). Interactions between Entodinium caudatum and an amino acid-fermenting bacterial consortium: fermentation characteristics and protozoal population in vitro. J Anim Sci Technol.

[83] Adachi K, Kawano H, Tsuno K (1999). Relationship between serum biochemical values and marbling scores in Japanese Black steers. J Vet Med Sci.

[84] Abe M, Iriki T, Tobe N, Shibui H (1981). Sequestration of holotrich protozoa in the reticulo-rumen of cattle. Appl Environ Microbiol.

[85] Punia BS, Leibholz J, Faichney GJ (1992). Rate of production of protozoa in the rumen and the flow of protozoal nitrogen to the duodenum in sheep and cattle given a pelleted diet of lucerne hay and barley. J Agric Sci.

[86] Sylvester JT, Karnati SKR, Yu Z, Newbold CJ, Firkins JL (2005). Evaluation of a real-time PCR assay quantifying the ruminal pool size and duodenal flow of protozoal nitrogen. J Dairy Sci.

[87] Eugène M, Archimède H, Sauvant D (2004). Quantitative meta-analysis on the effects of defaunation of the rumen on growth, intake and digestion in ruminants. Livest Prod Sci.

[88] Ivan M, Neill L, Entz T (2000). Ruminal fermentation and duodenal flow following progressive inoculations of fauna-free wethers with major individual species of ciliate protozoa or total fauna. J Anim Sci.

[89] Park T, Yang C, Yu Z (2019). Specific inhibitors of lysozyme and peptidases inhibit the growth of the rumen protozoan Entodinium caudatum without decreasing feed digestion or fermentation in vitro. J Appl Microbiol.

[90] Park T, Mao H, Yu Z (2019). Inhibition of rumen protozoa by specific inhibitors of lysozyme and peptidases in vitro. Front Microbiol.

[91] Shabat SKB, Sasson G, Doron-Faigenboim A (2016). Specific microbiome-dependent mechanisms underlie the energy harvest efficiency of ruminants. ISME J.

[92] Clemmons BA, Shin SB, Smith TPL (2021). Ruminal protozoal populations of angus steers differing in feed efficiency. Animals.

[93] de la Fuente G, Fondevila M, Belanche A, Morgavi D (2011). In vitro predation of pure bacterial species by rumen protozoa from monofaunated sheep, determined by qPCR. Options Mediterraneennes.

[94] Mosoni P, Martin C, Forano E, Morgavi DP (2011). Long-term defaunation increases the abundance of cellulolytic ruminococci and methanogens but does not affect the bacterial and methanogen diversity in the rumen of sheep. J Anim Sci.

[95] Park T, Cersosimo LM, Li W, Radloff W, Zanton GI (2021). Pre-weaning ruminal administration of differentially-enriched, rumen-derived inocula shaped rumen bacterial communities and co-occurrence networks of post-weaned dairy calves. Front Microbiol.

[96] Park T, Cersosimo LM, Radloff W, Zanton GI, Li W (2022). The rumen liquid metatranscriptome of post-weaned dairy calves differed by pre-weaning ruminal administration of differentially-enriched, rumen-derived inocula. Anim Microbiome.

[97] Bu D, Zhang X, Ma L (2020). Repeated inoculation of young calves with rumen microbiota does not significantly modulate the rumen prokaryotic microbiota consistently but decreases diarrhea. Front Microbiol.

[98] Gong J, Qing Y, Zou S (2016). Protist-bacteria associations: Gammaproteobacteria and Alphaproteobacteria are prevalent as digestion-resistant bacteria in ciliated protozoa. Front Microbiol.

[99] Pope PB, Smith W, Denman SE (2011). Isolation of Succinivibrionaceae implicated in low methane emissions from Tammar wallabies. Science.

[100] Indugu N, Vecchiarelli B, Baker LD, Ferguson JD, Vanamala JKP, Pitta DW (2017). Comparison of rumen bacterial communities in dairy herds of different production. BMC Microbiol.

[101] Onodera R, Henderson C (1980). Growth factors of bacterial origin for the culture of the rumen oligotrich protozoon, Entodinium caudatum. J Appl Bacteriol.

[102] Wang L, Abu-Doleh A, Plank J (2019). The transcriptome of the rumen ciliate Entodinium caudatum reveals some of its metabolic features. BMC Genomics.

[103] Findley SD, Mormile MR, Sommer-Hurley A (2011). Activity-based metagenomic screening and biochemical characterization of bovine ruminal protozoan glycoside hydrolases. Appl Environ Microbiol.

[104] Williams CL, Thomas BJ, McEwan NR, Rees Stevens P, Creevey CJ, Huws SA (2020). Rumen protozoa play a significant role in fungal predation and plant carbohydrate breakdown. Front Microbiol.

